# Integrative Bioinformatics approaches to therapeutic gene target selection in various cancers for Nitroglycerin

**DOI:** 10.1038/s41598-021-01508-8

**Published:** 2021-11-11

**Authors:** Jayaprakash Chinnappan, Akilandeswari Ramu, Vidhya Rajalakshmi V., Akil Kavya S.

**Affiliations:** Anthropology and Health Informatics Lab, Department of Bioinformatics, Bharathiar University, Coimbatore, Tamil Nadu India

**Keywords:** Computational biology and bioinformatics, Drug discovery, Systems biology, Diseases, Oncology

## Abstract

Integrative Bioinformatics analysis helps to explore various mechanisms of Nitroglycerin activity in different types of cancers and help predict target genes through which Nitroglycerin affect cancers. Many publicly available databases and tools were used for our study. First step in this study is identification of Interconnected Genes. Using Pubchem and SwissTargetPrediction Direct Target Genes (activator, inhibitor, agonist and suppressor) of Nitroglycerin were identified. PPI network was constructed to identify different types of cancers that the 12 direct target genes affected and the Closeness Coefficient of the direct target genes so identified. Pathway analysis was performed to ascertain biomolecules functions for the direct target genes using CluePedia App. Mutation Analysis revealed Mutated Genes and types of cancers that are affected by the mutated genes. While the PPI network construction revealed the types of cancer that are affected by 12 target genes this step reveals the types of cancers affected by mutated cancers only. Only mutated genes were chosen for further study. These mutated genes were input into STRING to perform NW Analysis. NW Analysis revealed Interconnected Genes within the mutated genes as identified above. Second Step in this study is to predict and identify Upregulated and Downregulated genes. Data Sets for the identified cancers from the above procedure were obtained from GEO Database. DEG Analysis on the above Data sets was performed to predict Upregulated and Downregulated genes. A comparison of interconnected genes identified in step 1 with Upregulated and Downregulated genes obtained in step 2 revealed Co-Expressed Genes among Interconnected Genes. NW Analysis using STRING was performed on Co-Expressed Genes to ascertain Closeness Coefficient of Co-Expressed genes. Gene Ontology was performed on Co-Expressed Genes to ascertain their Functions. Pathway Analysis was performed on Co-Expressed Genes to identify the Types of Cancers that are influenced by co-expressed genes. The four types of cancers identified in Mutation analysis in step 1 were the same as the ones that were identified in this pathway analysis. This further corroborates the 4 types of cancers identified in Mutation analysis. Survival Analysis was done on the co-expressed genes as identified above using Survexpress. BIOMARKERS for Nitroglycerin were identified for four types of cancers through Survival Analysis. The four types of cancers are Bladder cancer, Endometrial cancer, Melanoma and Non-small cell lung cancer.

## Introduction

Cancer is a terminal disease, triggered by uncontrolled cell growth known as tumor, and characterized by absence of apoptotic nature of the cells^1^. Recent studies have estimated 18.1 million new cancer cases (17 million, excluding non-melanoma skin cancer) and 9.6 million cancer deaths (9.5 million excluding non-melanoma) occurring every year world over. Lung cancer is the most common and frequently diagnosed cancer, followed by prostate cancer (rare in women), colorectal cancer (in terms of incidence), liver cancer, and stomach cancer (in terms of mortality) in both sexes^2^. Breast cancer is the most frequently diagnosed cancer in women and the leading cause of cancer death, followed by colorectal and lung cancer (in incidence) and cervical cancer (in mortality). Nitroglycerin, also known as glyceryl nitrate, stimulates vasodilation and is therefore an effective medication for coronary vascular disease (CVD). It also functions as a normalizer in tumor proliferation^3^. According to a new report, Nitroglycerin has great potential for treating cancer^4^. Though other treatments exist for various types of cancer, they are not successful every time. Besides treatments are also accompanied by severe side effects. In addition Nitroglycerin has pro-apoptotic and anti-angiogenic effects on tumor cells. They also help in developing immunity against tumor. In one study, the safety profile and efficacy of Nitroglycerin is determined and administered with chemo-radiotherapy^5^.

In recent years, various studies in the field of multi-center genomics research from gene to system level and next-generation sequencing, have provided insight into various mechanisms particularly progression of cancer and other diseases. Disease-associated nsSNP (non-synonymous Single Nucleotide Polymorphism) and cancer-associated SNPs, RNA Binding Protein (RBP) were found to be involved in the development of various types of cancer. Various in silico approaches such as molecular dynamics simulation, machine learning, mutational analysis, etc., determined the factors affecting progression of cancer and thereupon to develop potential drug therapies^6–12^. Identifying potential biomarker of drug compounds is now an emerging field of research in Integrative Bioinformatics Analysis^13^. Integrative Bioinformatics focuses on the problems of data integration for life science. Gong et al., established an Integrative Bioinformatics Analysis study for identifying the potential targets of aspirin in SCLC.

This study adopted the now popular Integrative Bioinformatics Analysis approach, wherein we identified ligand based direct target prediction of Nitroglycerin from PubChem and SwissTargetPrediction DB for finding the efficacy of the drug. Network and Pathway analyses were done with STRING and Cytoscape. Cancer associated with all known target genes were identified in CluePedia. 12 direct target genes were identified and they were found to be associated with different kinds of cancers. Mutational Analysis was performed and 3 target genes among the 12 direct target genes were found to be mutated. These were commonly associated with four types of cancers viz Bladder cancer, Endometrial cancer, Melanoma and Non-small cell lung cancer. These three mutated genes were selected for further study. The three identified genes were EGFR, HRAS and MAPK3. They were screened using “cBioPortal”. Further in OncoPrint genomic alteration frequency analysis revealed that the 3 genes were mutated in all four cancer types. Interconnected Genes associated with the 3 identified mutated target genes were predicted. Differential Expression of Gene Analysis (DEG Analysis) was performed using GEO2R to find upregulated and downregulated characteristics of the GEO datasets for four types of cancers. Pathway and Gene Ontology analyses were performed on the co-expressed genes. Survival analysis was performed using SurvExpress to validate the range of risk of the co-expressed genes. Finally, 4 genes were found as the potential biomarkers of Nitroglycerin.

## Materials and methodology

**Figure 1:**
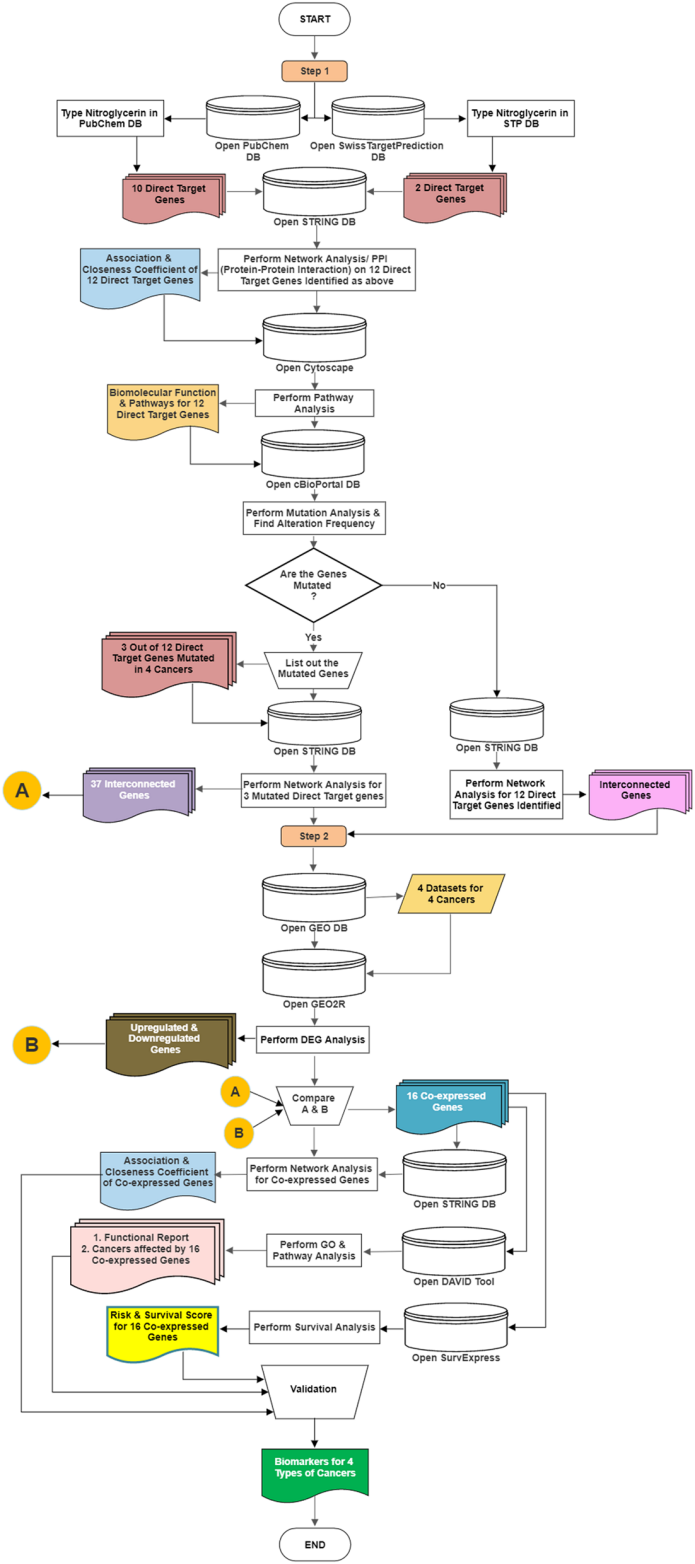
Flowchart of our research process.

Free version of the Flow Chart Creator [https://www.smartdraw.com] was used for getting the blunder free Flow Chart image using SmartDraw. SmartDraw's flowchart maker includes templates, tools, and symbols to make flowcharts easy and fast. Templates can be copied to MSOffice and Google apps from this flowchart App. Joint Photographic Experts Group (.jpeg) picture was transferred for better visualization. The flowchart is given in Fig. 1. This flowchart explains the overall methodology and details of databases and tools used.

### Identification of direct target genes

Target identification is the major process of identifying the direct and indirect molecular targets such as protein or nucleic acid (macro molecule). In bioinformatics, target identification is the process of finding the efficacy of a pharmaceutical/natural drug. In our study, direct target genes have been identified using Integrative Bioinformatics practice (drug based direct target). A total of 12 genes were identified as direct drug target for Nitroglycerin, 10 genes were obtained from the PubChem Database [https://pubchem.ncbi.nlm.nih.gov/] and 2 from SwissTargetPrediction [http://www.swisstargetprediction.ch/]. In PubChem Database Nitroglycerin targets revealed comprehensive outcome evaluation. Applying ‘Drug-Gene Interactions’, 10 target genes were obtained. SwissTargetPrediction allowed to estimate the most probable macromolecular targets of a small bioactive molecule. A combination of two dimensional and three dimensional similarity with a library of 3,70,000 known active proteins on more than 3 thousand proteins from different species are available^14^. SwissTargetPrediction genes were found in *Homo sapiens* after submitting the SMILES format of Nitroglycerin.

### Network and pathway analysis

Protein–Protein Interactions (PPIs) network functions as regulatory nodes in many cell-signalling networks associated with cancer's "hallmarks". A number of PPIs that are closely linked to cell signalling and cell survival have been identified and validated as cancer biomarkers, and they have become the subject of interest in academic and industry circles for drug discovery programs^15^. STRING (Search Tool for Interacting Genes Retrieval) [https://string-db.org/] is an online tool to construct and analyse protein–protein interaction network of Nitroglycerin, with not more than 20 interactors at the first, and second shells set as the cut off area. Cytoscape is a free software platform for visualizing molecular interaction networks and biological pathways as well as combining them with annotations, gene expression profiles, and other state data. CluePedia tool is a plugin with Cytoscape^16^ was used to identify crucial modules for further analysis.

1. STRING assessed the Nitroglycerin pathway, which was then checked and visualized by CluePedia^17^.
2. Pathways were identified for every known direct target genes from KEGG pathway using CluePedia.
3. Cancers associated with all known target genes were identified using CluePedia. Of the twelve target genes three target genes that were mutated were found to be associated with four types of cancer viz Bladder cancer, Endometrial cancer, Melanoma and Non-small cell Lung cancer. These were selected for further analysis.

### Mutation analysis of direct target genes

For tumors, mutation analysis is a common procedure to detect therapeutic sensitizing and resistant mutations^18^. It allows for a more precise prognosis and diagnosis, as well as personalized therapies tailored to meet specific tumor profile of each patient^19^. “cBioPortal” (c Bio Cancer Genomics Portal) [http://cbioportal.org] is a free, online and open platform for exploring multidimensional cancer genomics data. ‘OncoPrint’ is a tool that can show tumor sample alterations in gene arrays. cBioPortal revealed fifteen datasets for 12 direct target genes. A comparison of datasets and 12 direct target genes revealed mutation in different types of cancer influenced by 12 target genes individually. Identification and visualization of the listed 12 direct target genes and their associated cancer types revealed that 3 genes affected 4 types of cancer namely Bladder cancer, Endometrial cancer, Melanoma and Non-small cell lung cancer. The three Nitroglycerin-associated target genes are EGFR, HRAS and MAPK3. Both mutations and the genomic alteration frequency within the selected cancers were ascertained using cBioPortal.

### Prediction of interconnected genes for three mutated genes

The 3 mutated target genes (EGFR, HRAS and MAPK3) found in mutation analysis were input into PPI network. Using interactions with a high confidence score, genes which were associated (interconnected) with the three mutated target genes of Nitroglycerin were obtained with the help of STRING database (version 11). The interconnected genes were identified by a step-by-step process. Maximal groups/cliques were extracted from the PPI network. Each clique and the hub genes was notified by key pathways. 39 Interconnected genes were obtained. Of the 39, genes were found to be duplicated and hence 37 genes were chosen for further studies.

### Microarray data information and DEG analysis

NCBI-Gene Expression Omnibus (GEO) is a freely available database of gene/microarray profiles and next-generation sequencing (NGS) data. Microarray datasets were downloaded from GEO for four prominent cancers as follows:- Bladder cancer (GSE7476) (Last update date: March 25, 2019)^20^, Endometrial cancer (GSE17025) (Last update date: February 07, 2020)^21,22^, Melanoma (GSE35389) (Last update date: March 25, 2019)^23^ and Non-small cell lung cancer (GSE32989) (Last update date: May 27, 2020)^24,25^. These four datasets (pertaining to four types of cancers) were chosen for further DEG Analysis.

Differential Expression of Gene (DEG) Analysis was used to study and compare the gene expression between normal sample and diseased sample. Criteria for Upregulated and Downregulated genes in cancers were defined using GEO2R tool (a cancer microarray database and web-based data-mining platform)^26^. Cancer type (Bladder cancer, Endometrial cancer, Melanoma and Non-small cell lung cancer) and analysis type (‘cancer vs. normal’ analysis) were selected as the filters. We defined the corresponding adjusted *p* value for genes. An adjusted *p* value < 0.05 and logFC (fold change) ≥ 1 for Upregulated or ≤ -1 cutoff criteria for Downregulated genes and were also defined^27^.

### Finding co-expressed genes of target genes

A manual comparison of 37 Interconnected Genes found after Mutation Analysis (step 1 as per flowchart) with Upregulated/Downregulated genes found from DEG Analysis (Step 2 as per flowchart) was done to ascertain 16 co-expressed genes which in turn were used for further analysis.

### Network analysis (linkage) and validation of co-expressed genes

Network analysis in STRING was used to:

- Predict linkage (relationship) between co-expressed genes
- Find the degree of closeness particularly high closeness between co-expressed genes
- High betweenness prediction of the co-expressed genes

Cytoscape Network Analyser was used to validate the network analysis.

### GO and pathway enrichment analysis of co-expressed genes

The functions and pathway enrichment of candidate DEGs were analysed using DAVID tool version 6.8 [http://david.ncifcrf.gov/]. Gene ontology is a bioinformatics resource that provides information about gene product function. DAVID provides a comprehensive set of functional annotation tools to investigate large list of genes. It also helps in analysing biological roles of genes. It is used to perform GO and KEGG pathway enrichment analyses of differential expression of genes. Using GO study, functions of possible co-expressed genes of Nitroglycerin in four cancers were identified^28^.

### Survival analysis and validation

Survival analysis is used to analyse the probability distribution of survival of biological organisms. Survival analysis was performed using SurvExpress tool (An Online Biomarker Validation Tool and Database for Cancer Gene Expression Data Using Survival Analysis)^29^. SurvExpress is a large, versatile, and fast tool available freely on the Net. The input for SurvExpress is a list of co-expressed genes.

## Results

### Drug target identification

The genes that interacted with Nitroglycerin drug were retrieved and identified from PubChem database^30^ and SwissTargetPrediction database (Table 1).

**Table 1:** PubChem and SwissTargetPrediction for direct target genes of Nitroglycerin.

NCBI GENE_ID	GENE_NAME	INTERACTION CLAIM SOURCE	INTERACTION TYPE	DRUG NAME
**PubChem**				NITROGLYCERIN
2982	GUCY1A3	Chembl	Activator	
2944	GSTM1	NCI	Functionally unknown	
5595	MAPK3	NCI	Functionally unknown	
3091	HIF1A	MyCancerGenomeClinicalTrial	Inhibitor	
1956	EGFR	TTD	Functionally unknown	
3265	HRAS	NCI	Functionally unknown	
4881	NPR1	DrugBank, TEND, TdgClinicalTrial	Agonist	
2983	GUCY1B3	ChemblInteractions	Activator	
2977	GUCY1A2	ChemblInteractions	Activator	
2974	GUCY1B2	ChemblInteractions	Activator	
**SwissTargetPrediction**				
2936	GSR	GeneCards	Activator	
1610	DAO	GeneCards	Functionally unknown	

This shows direct target genes interactions with respect to Drug, Gene Name, Gene ID, Interaction claim source and Interaction type. Gene names are official gene symbols that are unique identifiers. Interaction claim source is the interaction taken from other available chemical compound databases. Interaction type is a function of inhibitor for target.

Depending on the degree of interaction, identified genes were segregated as functionally known Activator (GUCY1A3, GUCY1B3, GUCY1A2, GUCY1B2 and GSR), Inhibitor (HIF1A), and Agonist (NPR1). Apart from the previously mentioned genes, functionally unknown genes were also identified. Examples of Functionally unknown genes are GSTM1, MAPK3, EGFR, HRAS, DAO. Although, GSTM1 was identified as a functionally unknown gene, it was identified as Suppressor^31^. Similarly functionally unknown gene EGFR is activated by autocrine or paracrine growth factors in some tumors^32^. The functionally unknown EGFR gene is also responsible for gene amplification and is found to be prevalent in several types of cancer. It also plays an important role in abnormal EGFR signalling^32^.

### Analysis of the connection between target genes and cancer

**Figure 2:**
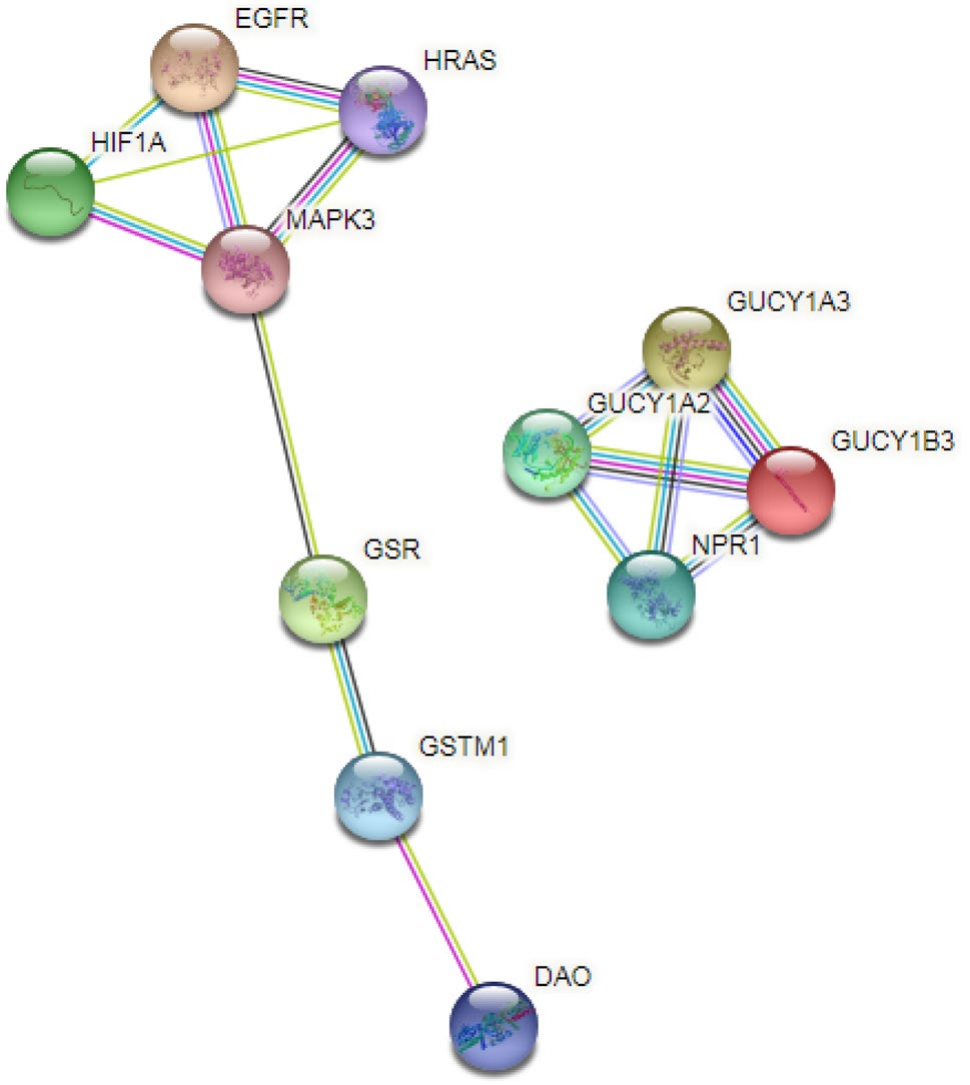
PPI network of Nitroglycerin target genes. Figure 1 shows that a group of genes EGFR, HRAS, MAPK3 and HIF1A were directly and indirectly connected with one another. Therefore, these genes were functionally linked and related. Another group of genes GUCY1A3, GUCY1A2, NPR1 and GUCY1B3 were functionally interconnected with one another.

STRING was used to develop PPI network and signalling pathways of genes with Nitroglycerin^33^ (Fig. 2). Interaction analyses show 11 nodes, 15 edges, 2.73 average node degree, 0.773 average local clustering coefficient and PPI enrichment *p* value of 0.000301. The predicted networks in all places in this work has significantly more acceptable interactions as per the reference value given in the STRING database (PPI enrichment *p* value = 1.0e−16).

**Figure 3:**
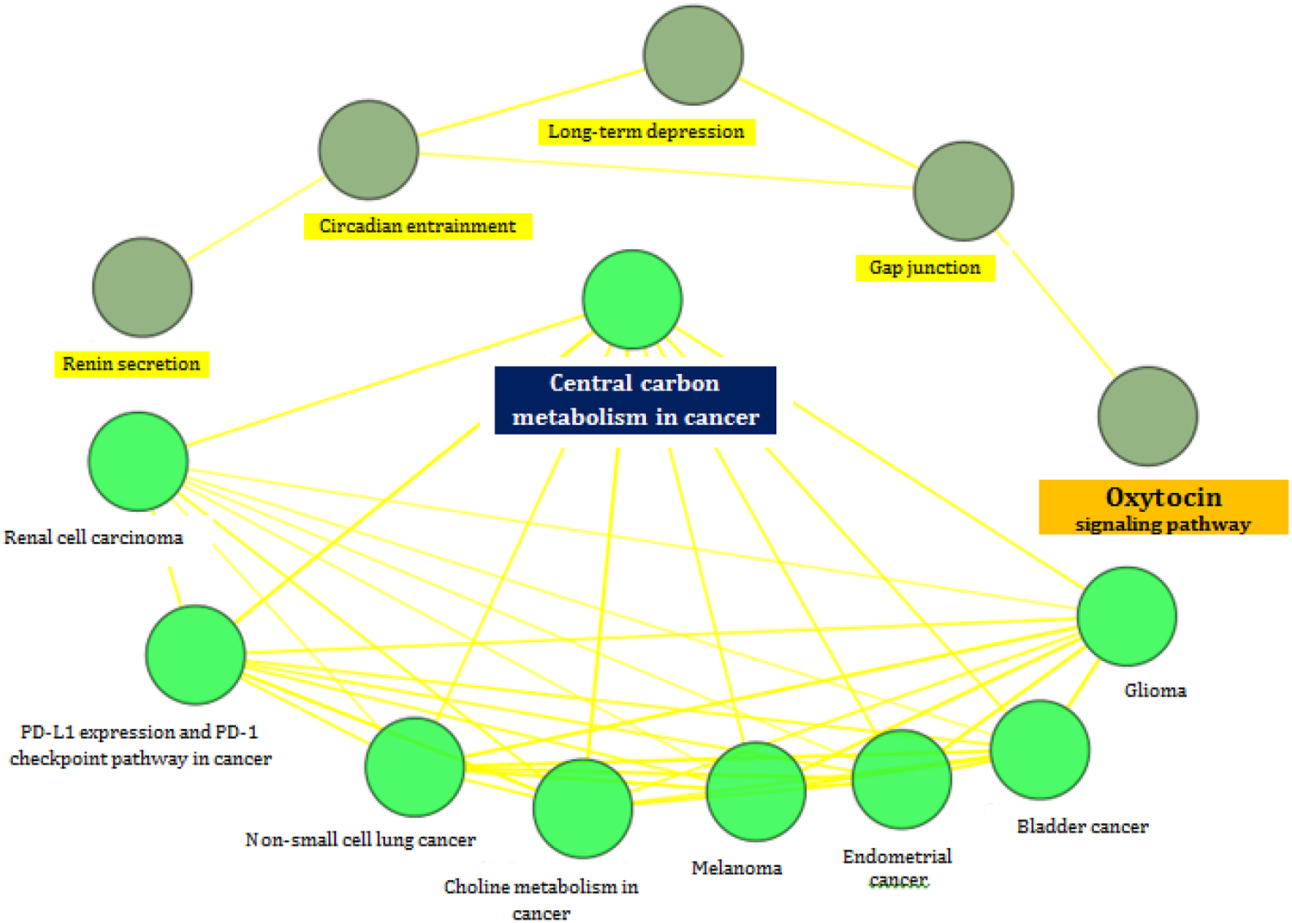
KEGG pathway Analysis for known target genes of Nitroglycerin. (i). diagram shows target genes and associated diseases^34^. Identification showed nine groups between target genes and disease connection such as: (1) *Glioma* (EGFR, HRAS, MAPK3), (2) *Bladder cancer* (EGFR, HRAS, MAPK3), (3) *Endometrial cancer* (EGFR, HRAS, MAPK3), (4) *Melanoma* (EGFR, HRAS, MAPK3), (5) *Choline Metabolism in cancer* (EGFR, HRAS, MAPK3, HIF1A), (6) *Non-small cell lung cancer* (EGFR, HRAS, MAPK3), (7) *PD-L1 expression and PD-1 checkpoint pathway in cancer* (EGFR, HIF1A, HRAS, MAPK3), (8) *Renal cell carcinoma* (H1F1A, HRAS, MAPK3), (9) *Central Carbon metabolism in cancer* (EGFR, HRAS, MAPK3, HIF1A). (ii). diagram shows the target genes has the connectivity in pathway: (1) *Oxytocin signalling pathway* (EGFR, GUCY1A1, GUCY1A2, GUCY1B1, HRAS, MAPK3, NPR1), (2) *Gap Junction* (EGFR, GUCY1A1, GUCY1A2, GUCY1B1, HRAS, MAPK3), (3) *Long-term depression* (EGFR, GUCY1A1, GUCY1A2, GUCY1B1, HRAS, MAPK3), (4) *Circadian entrainment* (EGFR, GUCY1A1, GUCY1A2, GUCY1B1, HRAS, MAPK3), and (5) *Renin secretion* (EGFR, GUCY1A1, GUCY1A2, GUCY1B1, NPR1).

The projected target genes were interlinked with cancers like Glioma, Bladder cancer, Endometrial cancer, Melanoma, Non-small cell lung cancer and Renal cell carcinoma. Choline Metabolism, PD-L1 expression and PD-1 checkpoint pathway in cancer and Central Carbon metabolism related to cancer interlinked with projected target genes were retrieved from the KEGG pathways analysis through Cytoscape (Fig. 3). The findings revealed that three Nitroglycerin genes (EGFR, HRAS and MAPK3) were found to be common in 4 types of cancers viz Bladder cancer, Endometrial cancer, Melanoma and Non-small cell lung cancer. Association of four cancers confirmed a positive correlation between 3 mutated genes of Nitroglycerin and the afore mentioned four cancers. Incidentally a positive correlation between 3 target genes of Nitroglycerin and four cancers was an unexpected outcome^15^.

### Analysis of genetic alteration in cancers

Mutational analysis was done to identify the genomic changes of 12 genes in various cancers. From the genomic changes identified above, genes with prominent expressions in cancers are identified. Prominent expression of genes as identified above and their associated cancers are identified. Genes having prominent expression are EGFR, HRAS and MAPK3. The cancers associated with the 3 genes as identified above are Bladder cancer, Endometrial cancer, Melanoma and Non-small cell lung cancer.

#### Mutational analysis in 4 cancers

**Figure 4:**
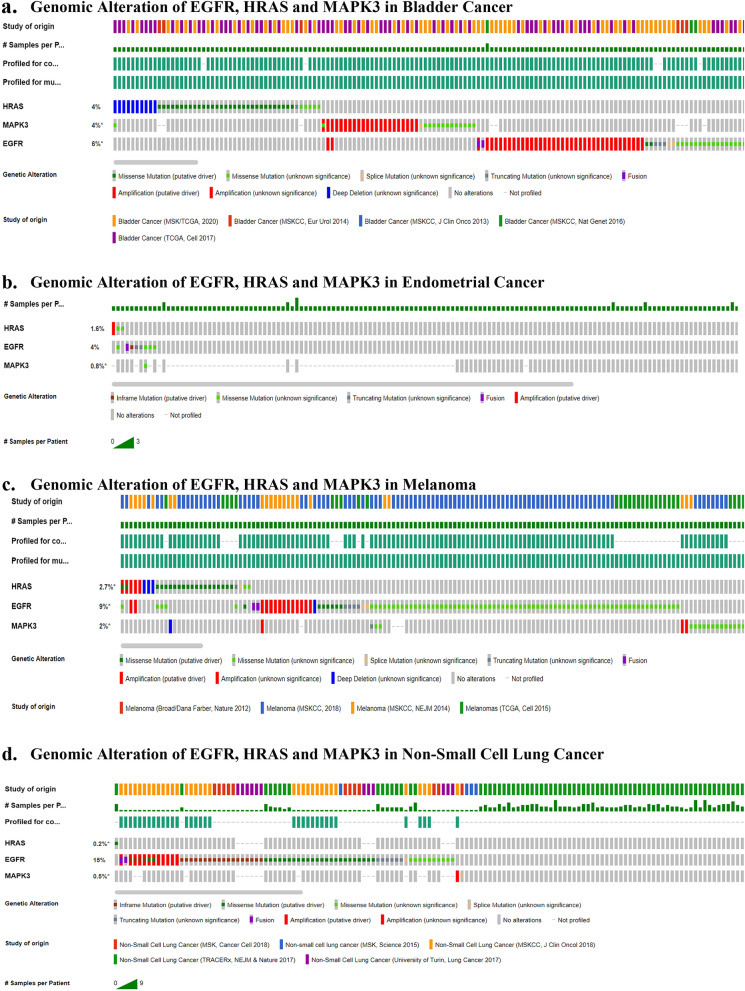
Genomic alteration of EGFR, HRAS and MAPK3 in all four cancer types. Green color denotes “missense mutation” of known significance, Light Green color denotes “missense mutation” of unknown significance, Yellow color denotes “Splice mutation”, Grey color denotes “Truncating mutation” of unknown significance, Violet color denotes “fusion”, Red color denotes “amplification” and Blue color denotes “deep deletion” of unknown significance.

cBioPortal was used to investigate the genomic changes of three Nitroglycerin genes (EGFR, HRAS and MAPK3) associated with respective cancers (Tables 2, 3). OncoPrint was used to show the most important alteration frequency of genes (Fig. 4).

**Table 2:** Position of gene (EGFR, HRAS, MAPK3) Mutations in cancers.

STUDY	PROTEIN CHANGE	GENE	CHR
**a. Bladder cancer**			
Bladder Cancer (MSK/TCGA, 2020), Bladder Cancer (TCGA, Cell 2017), Bladder Cancer (MSKCC, Nat Genet 2016), Bladder Cancer (MSKCC, EurUrol 2014)	E884K, T785S, R836H, V121F, Q390R, **F359L**, E59K, -402 fs, E736K, S380C, X901_splice, X762_splice, Q565E, E963Q, S174R, I715M, I886Tfs*9, EGFR-GRB10, RHBDD2-EGFR	EGFR	7
Bladder Cancer (MSK/TCGA, 2020), Bladder Cancer (TCGA, Cell 2017), Bladder Cancer (MSKCC, EurUrol 2014)	**G13D**, Q61L, **Q61K**, G13V, **G13R**, G12S, K117N, E162K, R149Gfs*23, D33N, E91K, S145*	HRAS	11
Bladder Cancer (MSK/TCGA, 2020), Bladder Cancer (TCGA, Cell 2017)	I182N, D335N, E18Q, R318W, R16I, V68L, E98K, E194Q, X11_splice	MAPK3	16
**b. Endometrial cancer**			
Endometrial Cancer (MSK, 2018)	D770_P772dup, I664Sfs*41, A237V, N466T, Q820*, R138I, V660M, **EGFR**-**intragenic**	EGFR	7
	T74A, Q25H	HRAS	11
	K72N	MAPK3	16
**c. Melanoma**			
Melanoma (MSKCC, 2018), Melanomas (TCGA, Cell 2015), Melanoma (MSKCC, NEJM 2014)	R252L, E114K, G983R, P589L, G796S, P753S, G729R, C264Y, S1045F, H47Y, V592I, S77F, N604D, R98Q, P694S, D1009N, P1178L, P100S, P622S, P644L, L1139F, **S306L**, P195L, R334C, S447F, D1152N, E967K, E829*, A419V, G63E, E922K, L541F, L101F, E319K, P512S, K737E, Q390K, Q982*, S364F, P934S, R958C, D303N, T594I, S116F, **F359L**, N234S, S227F, R574, P637S, P1019S, G901E, E1004K, S1028L, T909I, A118V, E1137K, P192S, P644S, D393N, G33N, **P733S**, S452F, P741S, A155V, H370Y, P243S, M825I, S220F, P100L, G131R, S169N, P411L, S912F, P272L, E245G, G575E, G322D, L1017F, P1073H, Q1095*, M137V, H418Y, A702V, P992S, N996S, P1059S, Q52*, E685K, A647V, L372H, X687_splice, **EGFR**-**intragenic**, **EGFR**-**intragenic** – **Archer**	EGFR	7
	Q61R, **G13D**, **Q61K**, G12D, **G13R**, G13C, G13S, G12N, G13N, A59V, A146T, E143K, A59Rfs*32, P174L, X38_splice	HRAS	11
	R87W, E362K, G102D, A303V, G374K, P328L, P169L, F185I, N140S, P336Q, C178R, R211W, L133Q, S159F, I89N, Q366*, G23R, P246F	MAPK3	16
**d. Non-small cell lung cancer**			
Non-Small Cell Lung Cancer (MSK, Cancer Cell 2018), Non-Small Cell Lung Cancer (University of Turin, Lung Cancer 2017), Non-Small Cell Lung Cancer (TRACERx, NEJM & Nature 2017), Non-Small Cell Lung Cancer (MSKCC, J ClinOncol 2018), Non-small cell lung cancer (MSK, Science 2015)	T790M, L858R, L861Q, G719A, E746_A750del, L747_S752del, L747_A750delinsP, S752_I759del, S768_D770dup, H773dup, N771_H773dup, P772_H773dup, D770_N771insY, V769_D770insSSV, L747P, R108K, E709A, T725M, T363A, Q787L, T725P, A864P, V774M, A839T, L707W, V765L, X210_splice, **S306L**, H870R, A13T, V616L, C1049R, R527W, R527W, S752F, L90F, L93I, L93F, **P733S**, A822T, E746Dfs*2, T751P, L438V, E746Nfs*2, L747*, K745Rfs*3, K745Rfs*3, E545*, A86T, Y299F, M111I, **EGFR**-**intragenic**	EGFR	7
Non-Small Cell Lung Cancer (TRACERx, NEJM & Nature 2017)	P34S	HRAS	11
Non-Small Cell Lung Cancer (MSK, Cancer Cell 2018)	X57_splice	MAPK3	16

This table shows that (a) in Bladder cancer 23 amino acids were mutated in EGFR Gene,12 amino acids were mutated in HRAS Gene and 23 amino acids were mutated in MAPK3 gene where positions mentioned in the table; (b) in Endometrial Cancer 8 amino acids were mutated in EGFR Gene where positions mentioned in the table, amino acid T was mutated to A at position 74 and amino acid Q was mutated to H at position 25 in HRAS Gene and amino acid K was mutated to N at position 72 of MAPK3 Gene; (c) in Melanoma 94 amino acids were mutated in EGFR Gene, 15 amino acids were mutated in HRAS Gene and 18 amino acids were mutated in MAPK3 gene as per positions mentioned in the table; (d) in Non-small cell lung cancer 52 amino acids were mutated in EGFR gene as per positions mentioned in the table, amino acid P was mutated to S at position 34 in HRAS Gene and amino acid X undergo splice mutation at position 57 in MAPK3 Gene.

**Table 3:** Type of gene alteration and alterations percentage.

Types of cancer	EGFR		HRAS		MAPK3	
	Gene alteration	Alterations percentage^a^	Gene alteration	Alterations percentage^a^	Gene alteration	Alterations percentage^a^
Bladder cancer	23 missense and 3 truncating mutation	6% (65/1028)	36 missense and 2 truncating mutation	4% (47/1125)	14 missense and 1 splice mutation	4% (36/879)
Endometrial cancer	4 missense, 2 truncating, 1 inframe and 1 Fusion mutation	4% (8/189)	2 missense mutation	1.6% (3/189)	1 missense mutation	0.8% (1/123)
Melanoma	99 missense, 4 truncating, and 2 Fusion mutation	9% (106/1129)	22 missense and 1 truncating mutation	2.7% (30/1129)	17 missense and 1 truncating mutation	2% (22/1091)
Non-small cell lung cancer	75 missense, 14 truncating, 23 inframe and 2 Fusion mutation	15% (72/472)	6 missense mutation	0.2% (1/432)	1 missense mutation	0.5% (2/375)

^a^Alterations percentage shows percentage of mutated sample out of total number of sample in all four cancer types.

#### Alteration frequency

**Figure 5:**
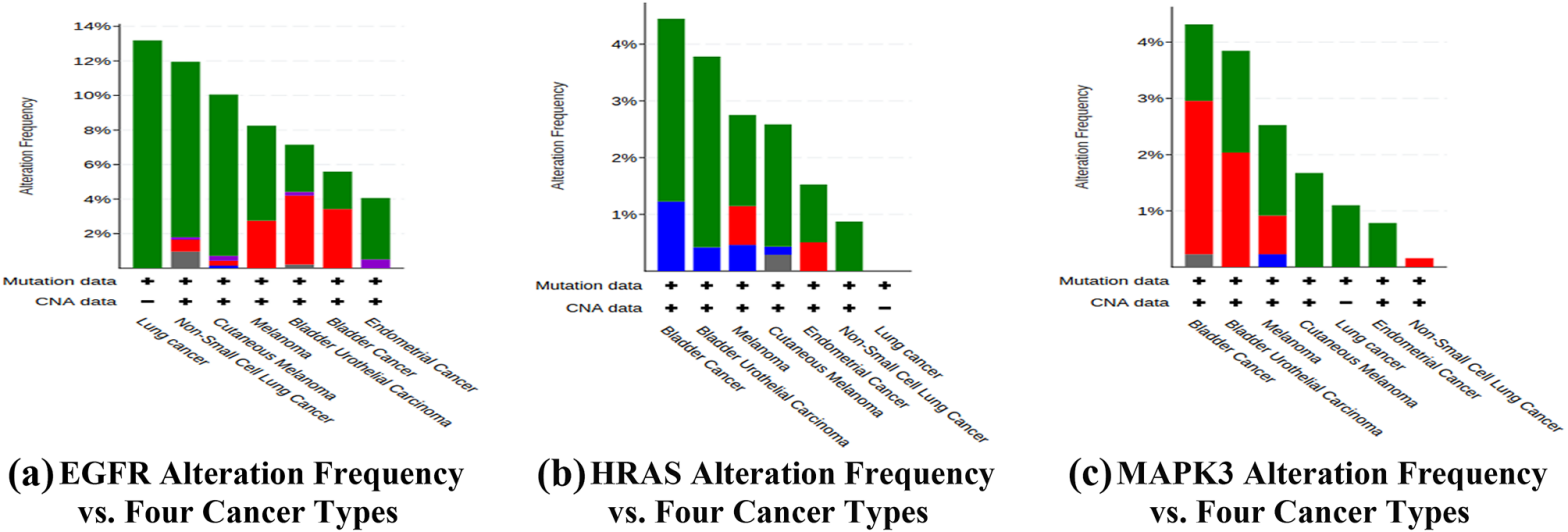
Alteration frequency versus four cancer types. The Y-axis denotes Alteration frequency and X-axis denotes cancer types. Bar diagram classified sample data based upon alteration frequency according to type of cancer. In the case of EGFR alteration frequency (**a**) occurred prominently in Lung Cancer and NSCLC. In the case of HRAS alteration frequency (**b**) occurred prominently in Bladder Cancer. In the case of MAPK3 alteration frequency (**c**) highly occurred prominently in Bladder Cancer.

15 studies consisting of 3,290 samples of Bladder cancer, Endometrial cancer, Melanoma and Non-small cell lung cancer were run in OncoPrint to find Alteration frequency of EGFR, HRAS and MAPK3 (Fig. 5). EGFR gene is prominently expressed in Lung Cancer and Non-Small Cell Lung Cancer. HRAS gene is prominently expressed in Bladder Cancer. MAPK3 is prominently expressed in Bladder Cancer. The following figure (Fig. 5) shows the Alteration frequency type of EGFR, HRAS and MAPK3 in four types of cancer. Alteration frequency type is expressed in colours:- Green indicates Mutation; Purple indicates Fusion; Red indicates Amplification; Blue indicates Deep deletion and Grey indicates Multiple Alterations.

### Prediction of interconnected genes for 3 mutated genes

**Figure 6:**
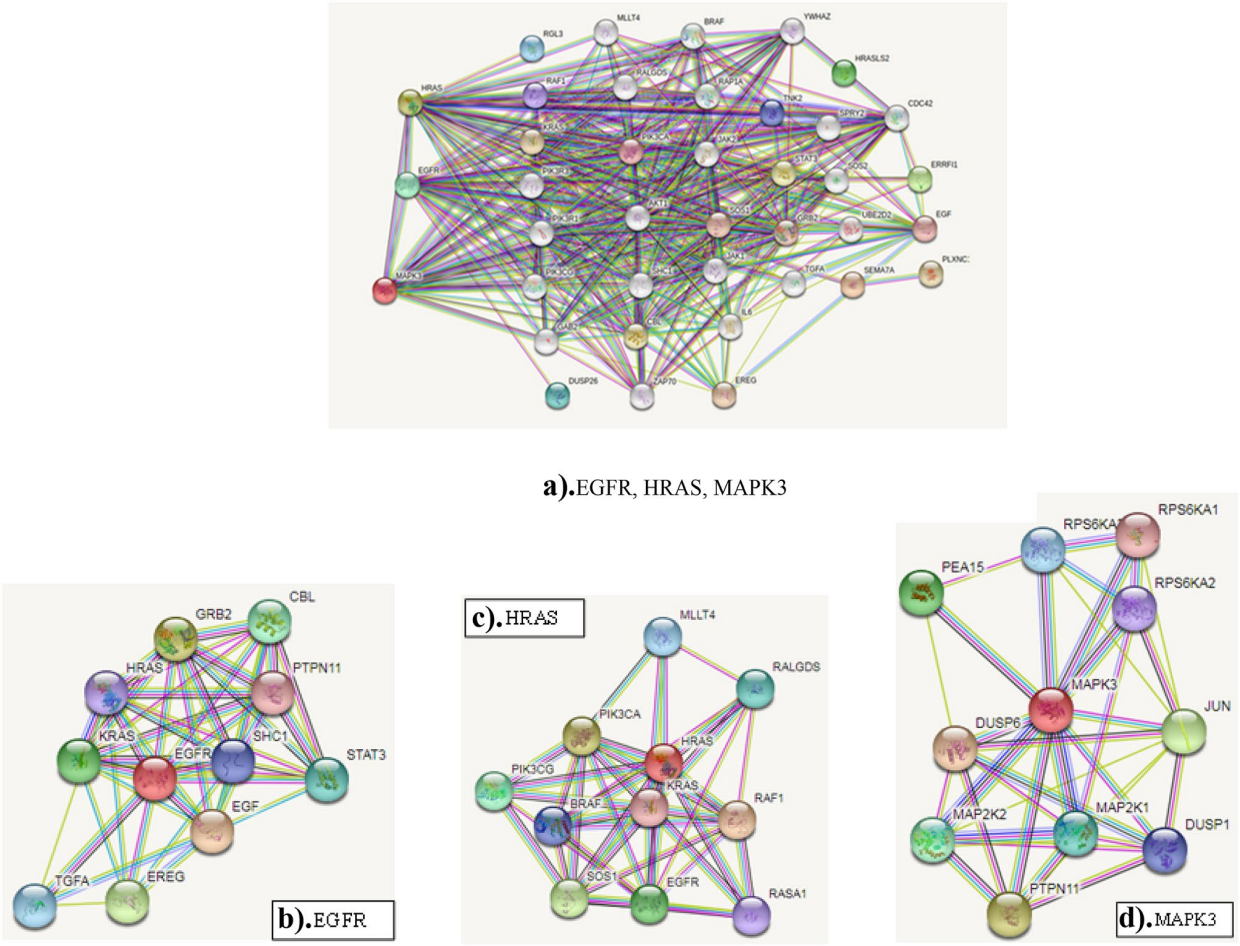
Protein–Protein Interaction of 39 associated genes. *Prediction of interconnected genes with EGFR, HRAS and MAPK3. The PPI analysis shows the thirty seven interconnected genes associated with EGFR, HRAS and MAPK3 targets. (**a**) shows the Interaction of 3 genes; (**b**,**c**,**d**) shows the Sample Specific Network for EGFR, HRAS and MAPK3.

Genes interconnected (network associated) with three target genes of Nitroglycerin were identified with the help of STRING database. Total of 39 associated genes (Fig. 6) were identified from protein–protein interaction analysis. The thirty nine associated genes are AKTI, BRAF, CBL, CDC42, DUSP26, EGF, EREG, ERRFI1, GAB2, GRB2, HRASLS2, IL6, JAK1, JAK2, KRAS, MLLT4, MLLT4, PIK3CA, PIK3CA, PIK3CG, PIK3R1, PIK3R3, PLXNC, RAA, RAF1, RALGDS, RAP1A, RGL3, SEMA7A, SHC1, SOS1, SOS2, SPRY2, STAT3, TGF, TNK2, UBE2D2, YWHAZ and ZAP70. MLLT4 and PIK3CA are duplicated. Hence duplication is removed and 39 genes are reduced to 37 genes.

These genes are directly or indirectly linked with three known target genes. Pathway analysis performed for these genes provide new avenues for Nitroglycerin therapeutic studies in cancers. Associate genes are mainly involved in pathways such as: EGFR tyrosine kinase inhibitor resistance, ErbB signaling pathway, Ras signaling pathway, Colorectal cancer, Non-small cell lung cancer, Glioma, Renal cell carcinoma, Pancreatic cancer, Phospholipase D signaling pathway and Chronic myeloid leukemia.

### DEG analysis

**Figure 7:**
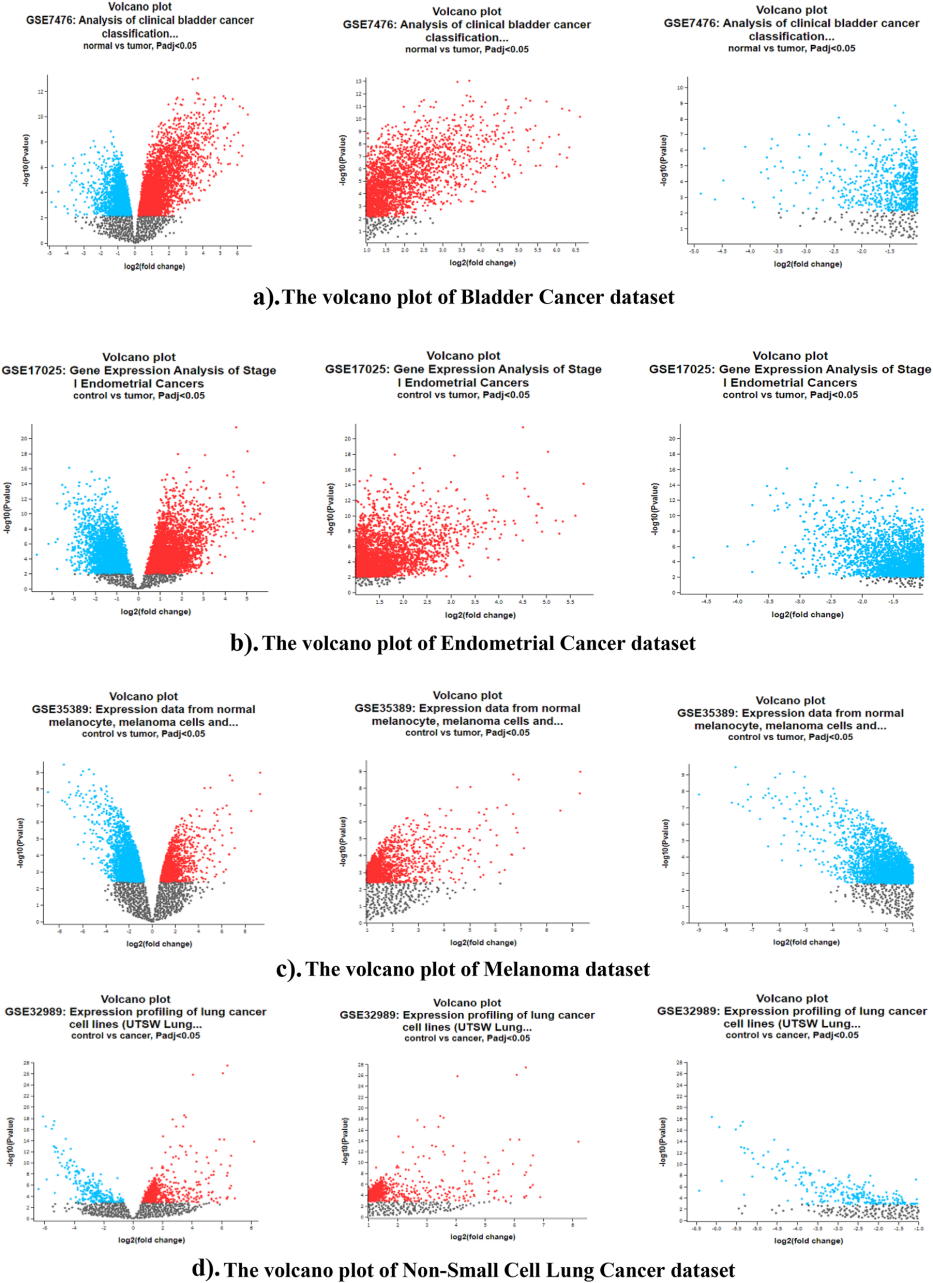
Volcano Plots of the four cancers. *Red color dots denote upregulated genes and blue color dots denote downregulated genes. Volcano plots were constructed using statistically significant genes only. Adjusted *p* value < 0.05 as the filtered upregulated DEGs based on logFC value (≥ 1) and downregulated DEGs based on logFC value (≤ -1).

DEG Analysis was done on four cancer datasets by comparing cancer samples with normal tissues in GEO2R tool (Fig. 7). The four types of cancer datasets are GSE7476 (Analysis of clinical bladder cancer classification according to microarray expression profiles), GSE17025 (Gene Expression Analysis of Stage I Endometrial Cancers), GSE35389 (Expression data from normal melanocytes, melanoma cells and their exosomes), GSE32989 (Expression profiling of lung cancer cell lines). Volcano plot was constructed for DEG analysis. The volcano plot shows the relationship between *p* values of a statistical test and the magnitude of fold change in terms of control versus cancer. The magnitude of fold change values denotes the extent to which genes were upregulated or downregulated. In volcano plot, the parameters of adjusted *p* value < 0.05 and logFC cutoff criteria ≥ 1 are upregulated and adjusted *p* value < 0.05 and logFC cutoff criteria ≤ -1 are downregulated were selected for our study.

### Co-expressed genes identification

Target genes and co-expressed genes will always have similar gene patterns and gene regulation^35,36^. The right approach to arrive at target biomarkers for Nitroglycerin is to adopt ways and means to find co-expressed genes (similar expression patterns).

By comparing the DEGs and interconnected genes with three target genes of Nitroglycerin, sixteen genes were found to be co-expressed (a possible Nitroglycerin therapeutic targets) in four types of cancers (Table 4). Result reveal Bladder cancer has five co-expressed genes. These are ERRFI1, IL6, PIK3R1 and SPRY2 which were found to be upregulated and YWHAZ which was found to be downregulated; Endometrial cancer has twelve genes. These are EGFR, ERRFI1, IL6, JAK2, PLXNC1, RGL3 which were found to be upregulated and CBL, CDC42, PIK3R3, STAT3, UBE2D2 and YWHAZ which were found to be downregulated; Melanoma has PLXNC1 as upregulated and TGFA as downregulated gene. Non-small cell lung cancer has GAB2 and PIK3R1 as two upregulated genes.

**Table 4:** Co-expressed gene identification in four types of cancer.

Gene symbol	Cancer	Count	Expression
ERRFI1, IL6, PIK3R1, SPRY2	Bladder	4	Up regulation
YWHAZ		1	Down regulation
EGFR, ERRFI1, IL6, JAK2, PLXNC1, RGL3	Endometrial	6	Up regulation
CBL, CDC42, PIK3R3, STAT3, UBE2D2, YWHAZ		6	Down regulation
PLXNC1	Melanoma	1	Up regulation
TGFA		1	Down regulation
GAB2, PIK3R1	Non-small cell lung	2	Up regulation

### Network analysis (linkage) and validation of co-expressed genes

**Figure 8:**
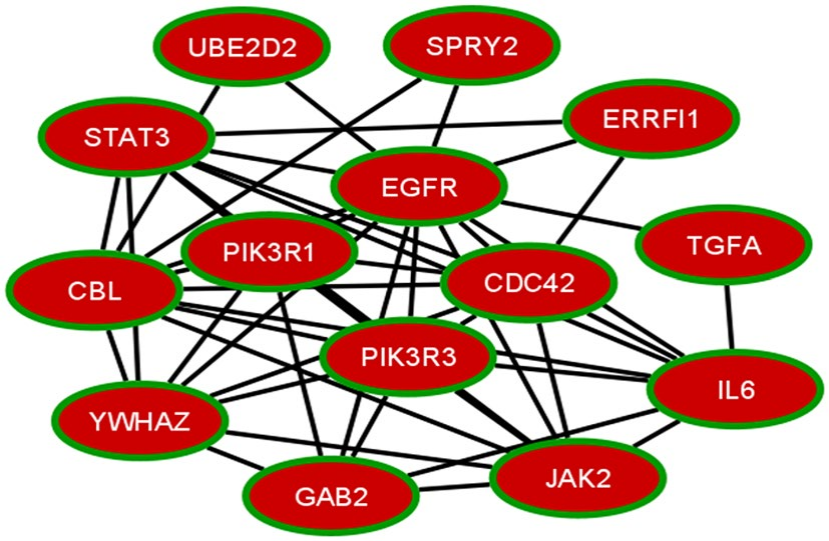
Linkage analysis of co-expressed genes.

Co-expressed genes linkage analysis of Bladder cancer, Endometrial cancer, Melanoma and Non-small cell lung cancer revealed nine potential genes viz EGFR, ERRFI1, GAB2, JAK2, IL6, PIK3R1, PLXNC1, RGL3, SPRY2 were upregulated and seven genes viz CBL, CDC42, PIK3R3, STAT3, TGFA, UBE2D2, YWHAZ were downregulated. Linkage was retrieved from STRING database (Fig. 8). It was validated by Network Analyser (Cytoscape). Genes PIK3R3, STAT3, JAK2, PIK3R1, EGFR, YWHAZ, UBE2D2, SPRY2 and TGFA have low average shortest path length. Centrality analysis examines the important node in the network, aiding drug target studies. Closeness of the Centrality estimates how much a node is close with other nodes. High closeness centrality genes in the network are EGFR, STAT3, PIK3R3, JAK2, PIK3R1 and CBL. High betweenness centrality genes are EGFR, IL6, STAT3, CDC42, JAK2, PIK3R3 and PIK3R1.

### GO and pathway analysis of co-expressed genes

KEGG pathway analysis and Gene Ontology analysis using DAVID database was performed for four types of cancer targets. KEGG pathway analysis showed that sixteen co-expressed genes were found to participate in pathways such as: Fc epsilon RI signaling pathway, Hepatitis B, Measles, Axon guidance, Ebb signaling pathway and PI3K-Akt signaling pathway (Table 5). GO analysis comprises three functional groups: (1) biological processes, (2) cellular components, and (3) molecular functions. In biological processes, majority of genes are involved in negative regulation of apoptotic process and phosphatidylinositol-mediated signaling. In cellular components, many of the genes are present in cytosol and cytoplasm. In Molecular function, majority of genes promote protein binding and protein kinase binding. Table 5 shows the pathways, process, location, function, disease and expression level for each individual gene.

**Table 5:** GO and pathway analysis of co-expressed genes.

Gene	KEGG	Biological process	Cellular component	Molecular function	Cancer	Expression
CBL	Pathways in cancer	Negative regulation of apoptotic process	Cytosol	Protein Binding	Endometrial	Down regulation
CDC42	Pathways in cancer	Unidentified	Cytosol	Protein Kinase Binding	Endometrial	Down regulation
EGFR	Pathways in cancer	Negative regulation of apoptotic process	Membrane raft	Protein Kinase Binding	Endometrial	Up regulation
ERRFI1	Unidentified	Negative regulation of collagen biosynthetic process	Cytosol	Protein Kinase Binding	Bladder, Endometrial	Up regulation
GAB2	Fc epsilon RI signaling pathway	Phosphatidylinositol-mediated signaling	Cytoplasm	Transmembrane receptor protein tyrosine kinase adaptor activity	Non-small cell lung	Up regulation
IL6	Hepatitis B, Pathways in cancer	Negative regulation of apoptotic process	Cytoplasm	Protein Binding	Bladder, Endometrial	Up regulation
JAK2	Measles	Unidentified	Cytosol	Protein Kinase Binding	Endometrial	Up regulation
PIK3R1	Hepatitis B, Fc epsilon RI signaling pathway	Negative regulation of apoptotic process, phosphatidylinositol-mediated signaling	Cytosol, Nucleus	Transmembrane receptor protein tyrosine kinase adaptor activity	Bladder, Non-small cell lung	Up regulation
PIK3R3	Pathways in cancer	Unidentified	Cytosol	Protein Binding	Endometrial	Down regulation
PLXNC1	Axon guidance	Cell adhesion	Semaphorin receptor complex	Protein Binding	Endometrial, Melanoma	Up regulation
RGL3	Unidentified	Unidentified	Unidentified	Unidentified	Endometrial	Up regulation
SPRY2	Unidentified	Negative regulation of apoptotic process	Cytosol	Protein Kinase Binding	Bladder	Up regulation
STAT3	Pathways in cancer	Negative regulation of apoptotic process	Cytosol	Protein Kinase Binding	Endometrial	Down regulation
TGFA	Ebb signaling pathway	Activation of MAPK activity	Golgi membrane	Glycoprotein binding	Melanoma	Down regulation
UBE2D2	Unidentified	Unidentified	Cytosol	Protein Binding	Endometrial	Down regulation
YWHAZ	Hepatitis B, PI3K-Akt signaling pathway	Negative regulation of apoptotic process	Cytosol	Protein Kinase Binding	Bladder, Endometrial	Down regulation

### Survival analysis (Kaplan–Meier plot and ROC curve)

**Figure 9:**
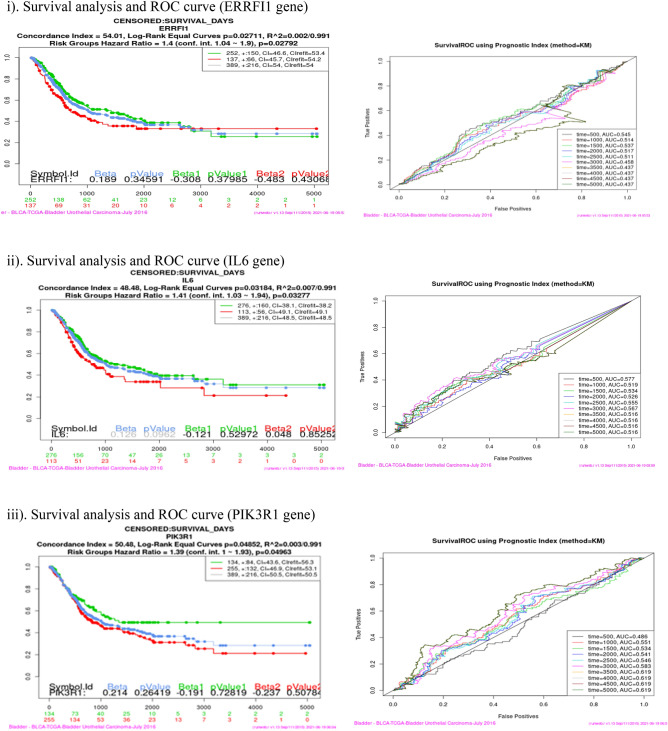

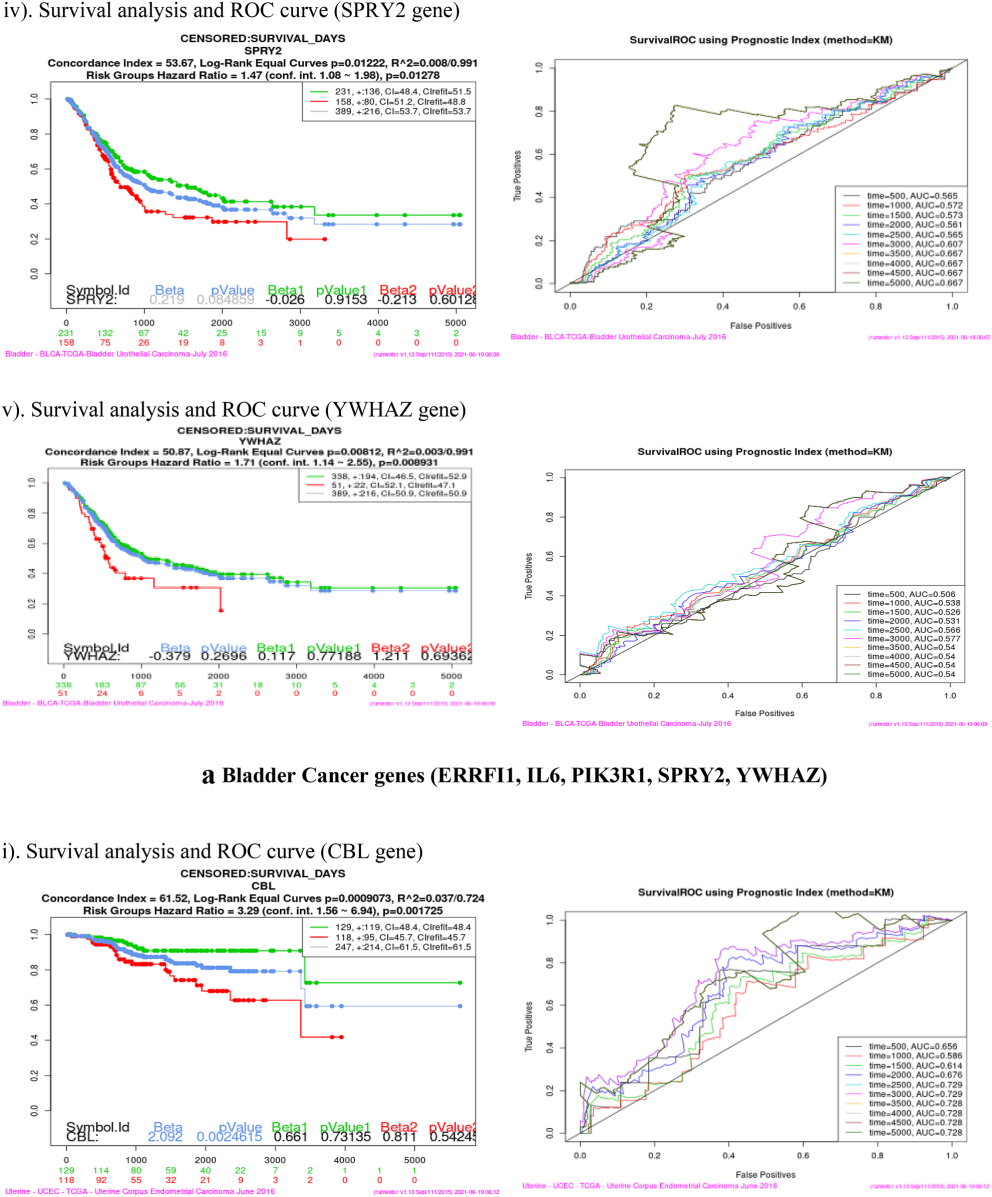

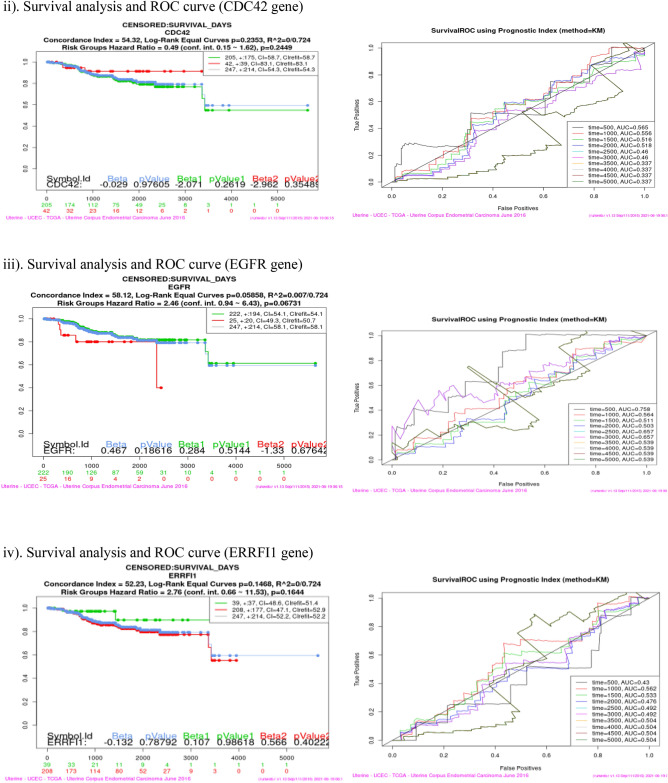

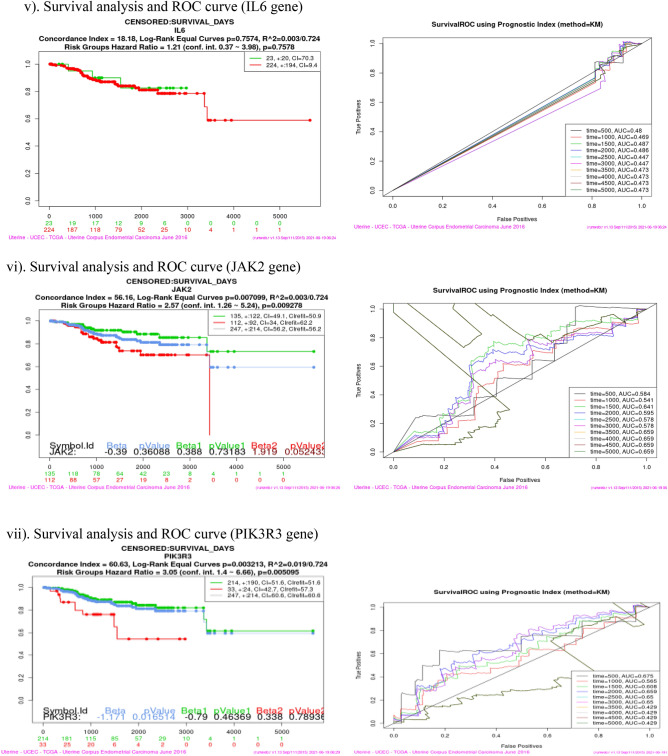

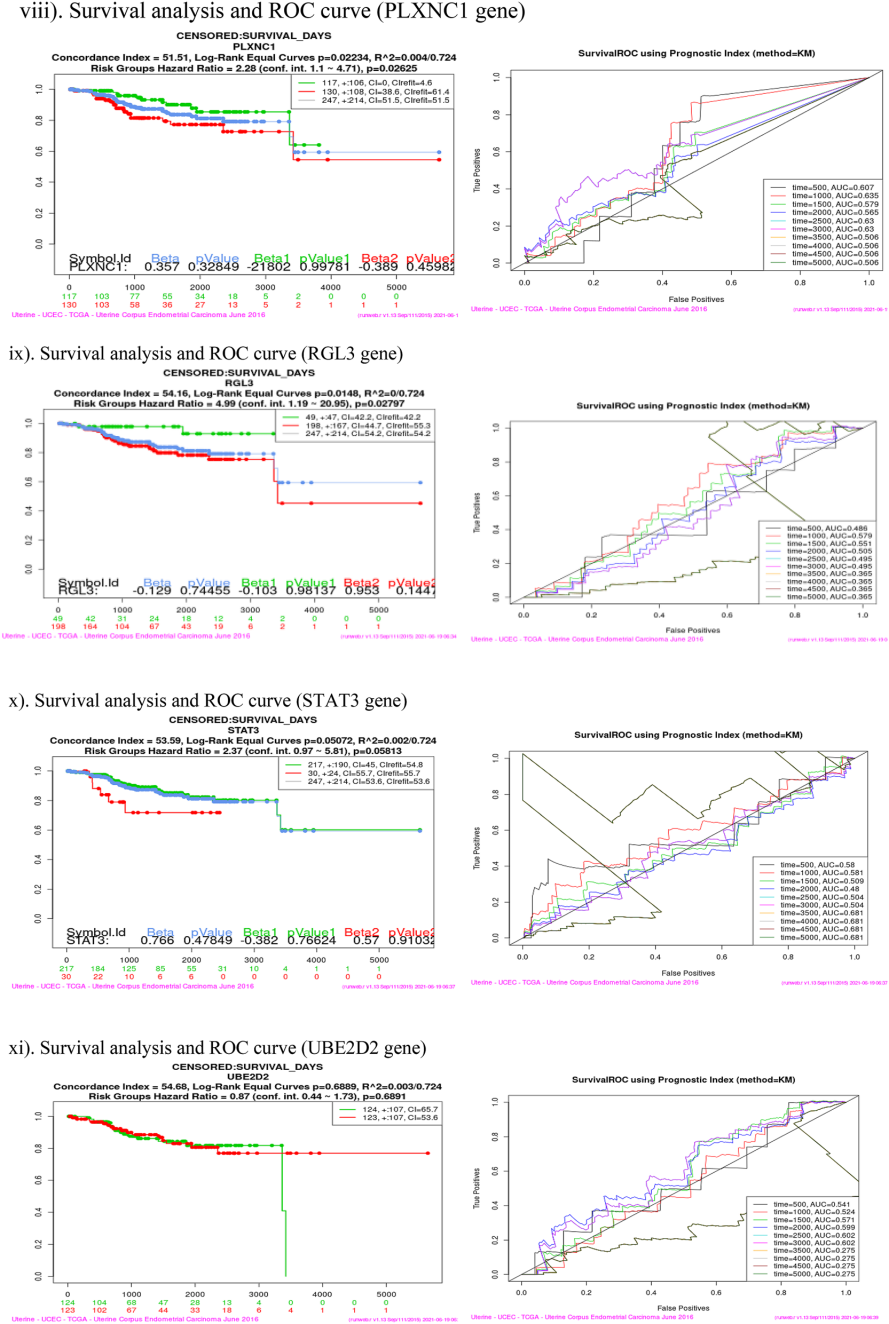

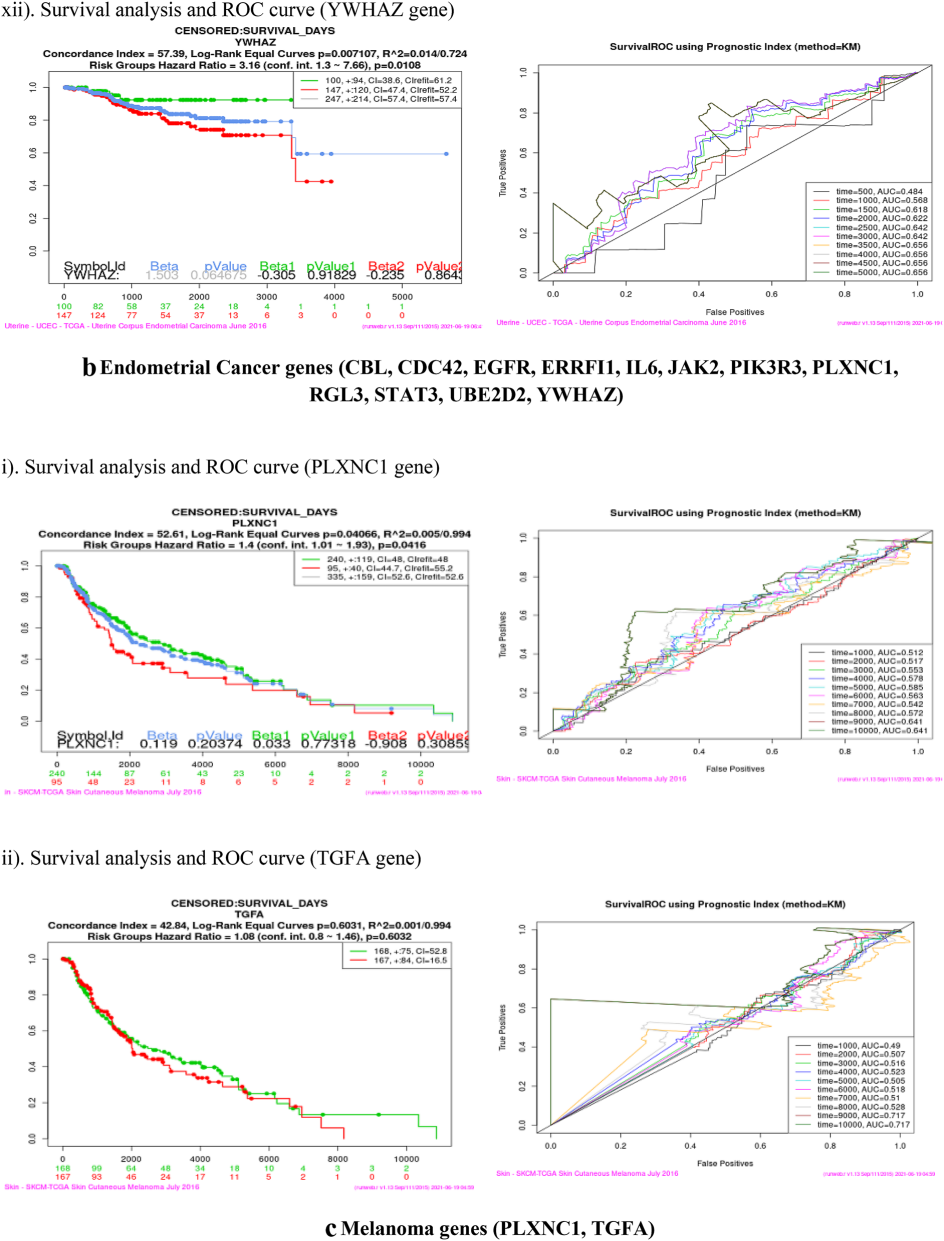

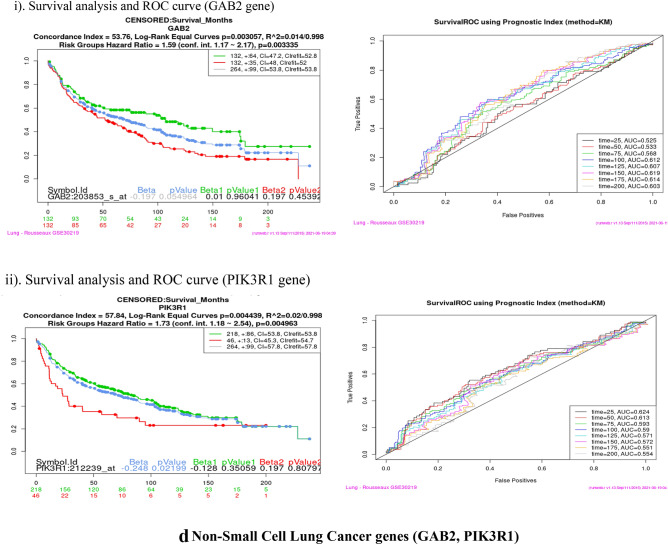
(**a**) Bladder cancer genes (ERRFI1, IL6, PIK3R1, SPRY2, YWHAZ). (**b**) Endometrial cancer genes (CBL, CDC42, EGFR, ERRFI1, IL6, JAK2, PIK3R3, PLXNC1, RGL3, STAT3, UBE2D2, YWHAZ). (**c**) Melanoma genes (PLXNC1, TGFA). (**d**) Non-small cell lung cancer genes (GAB2, PIK3R1).

Survival analysis was performed using SurvExpress tool for each of the sixteen co-expressed gene in four cancer dataset viz Bladder Cancer, Endometrial Cancer, Melanoma and Non-small lung cancer. This analysis helps to identify high risk of death and low survival of co-expressed genes (Fig. 9). Kaplan–Meier plot showed the Concordance Index (CI), *p* value for Survival Curve and Hazard Ratio for risk group. Higher CI values are associated with better prediction for Survival Curve. Survival risk curves are represented in green and red color for low and high risk respectively. The x-axis represents the time (in days) of the study. Hazard Ratio value (≥ 1) indicates high risk rate that leads to low survival rates. Risk group classification was validated by ROC (reoccurrence score) curve. Disease wise survival analysis of Hazard Ratio, *p* value and AUC (area under the ROC curve) values are mentioned Table 6.

**Table 6:** Survival probability and reoccurrence score.

Cancer	Co-expressed Genes	Hazard ratio	*P* value	AUC
Bladder	ERRFI1	1.4	0.2792	0.437
	IL6	1.41	0.03277	0.516
	PIK3R1	1.39	0.04963	0.619
	**SPRY2**	**1.47**	**0.01278**	**0.667**
	YWHAZ	1.71	0.008931	0.54
Endometrial	**CBL**	**3.29**	**0.001725**	**0.728**
	CDC42	0.49	0.2449	0.337
	EGFR	2.46	0.06731	0.539
	ERRFI1	2.76	0.1644	0.504
	IL6	1.21	0.7578	0.473
	JAK2	2.57	0.009278	0.659
	PIK3R3	3.05	0.005095	0.429
	PLXNC1	2.28	0.02625	0.506
	**RGL3**	**4.99**	**0.02797**	**0.365, 0.502**
	STAT3	2.37	0.05813	0.681
	UBE2D2	0.87	0.6891	0.275
	YWHAZ	3.16	0.0108	0.656
Melanoma	PLXNC1	1.4	0.0416	0.641
	**TGFA**	**1.08**	**0.6032**	**0.717**
Non-small cell lung	**GAB2**	**1.59**	**0.003335**	**0.603**
	PIK3R1	1.73	0.004963	0.554

Bold indicates the genes that have high risk rate and low survival rate. These genes are identified as Biomarkers for Nitroglycerin in this study.

Overall analysis revealed that RGL3 gene has high risk by the Hazard Ratio value but Survival ROC curve classification gave a less accurate score in prediction. Finally, SPRY2 (Bladder), CBL (Endometrial), TGFA (Melanoma) and GAB2 (Non-small cell lung) genes have high risk rate and low survival rate. These four genes are valid therapeutic targets (biomarkers) for Nitroglycerin from the co-expressed genes.

## Discussion

Cancer is the second deadliest disease in the world. Hence finding a novel drug is very important for reducing risk of death and increasing survival rate. However finding a novel drug by experimental approach of target identification is time consuming, and sometimes takes as long as even 12 years or more. On the other hand computational procedure for repurposing/finding a novel drug takes a very short time and at lower cost^37^.

In mutational analysis of four cancer sample studies, (bladder cancer, endometrial cancer, melanoma and non-small cell lung cancer) three genes whose frequency of genetic alterations were measured were found to be mutated. The three genes are EGFR, HRAS and MAPK3, Proteins within the gene are responsible for gene mutation. Mutation of proteins in two Genes EGFR and HRAS were found responsible for mutations in multiple cancers. Mutation of proteins in one gene MAPK3 was found to be unique to each type of cancer. Mutated proteins responsible for multiple cancers are F359L, P733S, S306L, G13D, G13R and Q61K. We found that **EGFR gene** that caused *intragenic mutation* (Gene-EGFR, chr-7 and type-Fusion) occurred in three types of cancers viz Endometrial Cancer (MSK, 2018), Melanoma (MSKCC, 2018) and Non-Small Cell Lung Cancer (MSKCC, J ClinOncol 2018); *F359L mutation* (Gene-EGFR, position-359(F-L), chr-7, type-missense) occurred in Bladder Cancer (TCGA, Cell 2017; https://www.cancer.gov/tcga) and Melanoma (MSKCC, 2018); *P733S mutation* (Gene-EGFR, position-733(P-S), chr-7, type-missense) occurred in Melanoma (MSKCC, 2018) and Non-Small Cell Lung Cancer (University of Turin, Lung Cancer 2017); *S306L mutation* (Gene-EGFR, position-306(S-L), chr-7, type-missense) occurred in Melanoma (MSKCC, 2018) and Non-Small Cell Lung Cancer (MSKCC, J ClinOncol 2018); **Gene HRAS**: *G13D mutation* (Gene-HRAS, position-13(G-D), chr-11, type-missense) occurred in Bladder Cancer (MSK/TCGA, 2020) and Melanoma (MSKCC, 2018); *G13R mutation* (Gene-HRAS, position-13(G-R), chr-11, type-missense) occurred in Bladder Cancer (TCGA, Cell 2017) and Melanoma (MSKCC, 2018); *Q61K mutation* (Gene-HRAS, position-61(Q-K), chr-11, type-missense) occurred in Bladder Cancer (TCGA, Cell 2017) and Melanoma (TCGA, Cell 2015). These proteins within the above discussed 3 genes can be taken for the therapeutic analysis. It is worth pointing out that since proteins within Gene EGFR and Gene HRAS affect multiple cancers as compared to proteins within Gene MAPK3 which are unique to each type of cancer, the cost benefit payoff ratio would be higher for Gene EGFR and Gene HRAS than for Gene MAPK3.

In the present study, we investigated the DEGs among four cancer data sets (cancer vs. normal). We examined a total of 20,529 DEGs, 2279 upregulated and 758 downregulated genes for Bladder cancer; 3238 upregulated and 2553 downregulated genes for Endometrial cancer; 52 upregulated and 69 downregulated genes for Melanoma; and 875 upregulated and 253 downregulated genes for Non-small cell lung cancer. While Nitroglycerin is commonly used for treatment of CVD patients and a number of studies have generally shown Nitroglycerin to be an antitumor agent, our research is at the minute level of genes. Our research dwells further into different gene targets such as Direct Target Genes, Mutated Genes, Interconnected Genes, Co-expressed genes, and finally Biomarkers of Nitroglycerin for the four cancers. Linkage analysis proved that co-expressed genes should have similar patterns in gene expression and gene regulation. GO and Pathway Analysis results confirmed that the co-expressed genes have a major role to play in many biological functions such as Protein Kinase Binding, Protein Binding, Glycoprotein binding, and Molecular Adaptor Activity of Transmembrane Receptor Protein Tyrosine Kinase. All these functions were disrupted by co-expressed genes in respective cancers. DEG analysis further revealed upregulation of genes SPRY2 (Bladder), and GAB2 (Non-small cell lung) and downregulation of genes CBL (Endometrial), TGFA (Melanoma) were associated with low survival rate and high risk of death as measured by survival probability and AUC score. This is corroborated by gene suppression of SPRY2^38^ that revealed distinct tumor suppressive roles in different cancer perspectives^39–41^. Further corroboration is obtained when the downregulation of SPRY2 caused significantly reduced cell proliferation/cell death^42^. Besides SPRY2 promoter plays an important role in ERK signaling and inhibition of several human cancers ^43,44^.

Studies reveal potential clinical impact of CBL gene on cancer immunotherapy. In our study gene CBL is identified as having high closeness among the 16 co-expressed genes in linkage analysis and predicted as downregulated gene via DEG analysis. Our study corroborates with an existing report that CBL positively regulates signal transduction which means it increases regulation (activate/upregulation) which in turn leads to reduction of complications in Endometrial cancer^45^. As per our findings, we suggest CBL as an apt target for Nitroglycerin and novel drug design against Endometrial cancer. As regards TGFA our study corroborates with overexpression of the gene, leading to non-progression of cancer^46^. Further corroboration is obtained with respect to study of TGFA expression in esophageal cancer^47^. Corroboration is obtained for GAB2 when suppression of the same reduces lymph node metastases and invasive cancer. GAB2 also seems to collaborate with other oncogenes linked to the progression of breast cancer, including the SRC family. Standard chemotherapy employs GAB2 as a potential gene target in treatment of GAB2-driven ovarian cancer. GAB2 is involved in signaling the growth of malignant tumors^48^.

## Conclusion

Though the identification of drug-gene interaction is significant in drug discovery approach, the cost overrun for experimental approaches is enormous. It is extensively time consuming and very challenging. To offset humongous cost overrun and time consuming practices, several computational practices including pharmacology of drugs and evaluating drug-target interactions are leading to the discovery/invention of potential Biomarkers for a drug. Our analyses based on the latter method of computational procedures promotes connecting the diseases with the drug-associated gene sets at a minimum cost and in quick time. Integrative Bioinformatics Analysis is a computational procedure which improves understanding the mechanism of drugs relating to cancer treatment in quick time and can be considered as versatile in explaining the concepts of drug-disease interaction.

Integrative Bioinformatics analyses helps to identify Biomarkers of Nitroglycerin drug in a few days which otherwise would have taken a few months or sometimes even a few years. After Survival Analysis we concluded that four genes (SPRY2-for Bladder cancer, CBL-for Endometrial cancer, TGFA-for Melanoma and GAB2 for Non-small cell lung cancer) were the Biomarkers for Nitroglycerin. The results of our research can now be used in experimental procedure to gain insight into the role of the identified Biomarkers in cancer treatment. The identified Biomarkers can also be used in further computational procedures.
